# Spectrum Lines

**Published:** 1869-08

**Authors:** 


Spectrum Lines.—At a late meeting of the American Institute, Professor E. C. Pickering, of the Massachusetts Institute of Technology, employed the following method to illustrate his paper on Spectrum Analysis. A sheet of black lace, one and a half feet broad, and three feet high, was suspended as a screen, and upon it was thrown a continuous spectrum from a magnesium light, arranged in the manner first developed for the electric light by Prof. Cook, of Cambridge, except that only one bisulphide of carbon prism was used. The spectrum covered the entire lace screen, the curvature of its lines being corrected by an opposite curvature of the opening through which the light passed. Upon this black lace were attached a number of white paper strips, so arranged as to occupy the places of the bright lines in seven different spectra, such as those of sodium, potassium, rubidium, cœsium, etc. These being illuminated by the variegated light of the continuous and broad spectrum, received and reflected each the color corresponding to its position, and therefore, (their adjustment being accurate,) that which actually belonged to the band which it represented. The light falling between these paper bands was not, of course, reflected, and the appearance, therefore, was in each case (as an actual spectrum), of bright-colored lines on a dark ground. When, however, as in the case of potassium, the nebula of Orion, etc., a faint, continuous spectrum is in fact combined with that of the bright lines, this also was represented by attaching, in the right places, bands of white lace, which reflected enough of the colored light to produce a hazy spectrum, admirably imitating that of the substances or bodies in question.—*Jour. Franklin Institute.*

